# MCP-1 Predicts Recurrent Cardiovascular Events in Patients with Persistent Inflammation

**DOI:** 10.3390/jcm10051137

**Published:** 2021-03-09

**Authors:** Luis M. Blanco-Colio, Nerea Méndez-Barbero, Ana María Pello Lázaro, Álvaro Aceña, Nieves Tarín, Carmen Cristóbal, Juan Martínez-Milla, Óscar González-Lorenzo, José Luis Martín-Ventura, Ana Huelmos, Carlos Gutiérrez-Landaluce, Marta López-Castillo, Andrea Kallmeyer, Ester Cánovas, Joaquín Alonso, Lorenzo López Bescós, Jesús Egido, Óscar Lorenzo, José Tuñón

**Affiliations:** 1 Vascular Research Lab, IIS-Fundación Jiménez Díaz, 28040 Madrid, Spain; lblanco@fjd.es (L.M.B.-C.); nerea.mendez@quironsalud.es (N.M.-B.); JLMartin@fjd.es (J.L.M.-V.); 2 Centro de Investigación Biomédica en Red de Enfermedades Cardiovasculares (CIBERCV), Instituto de Salud Carlos III, 28029 Madrid, Spain; 3 Department of Cardiology, IIS-Fundación Jiménez Díaz, 28040 Madrid, Spain; ampello@quironsalud.es (A.M.P.L.); aacena@fjd.es (Á.A.); juan.mmilla@quironsalud.es (J.M.-M.); ogonzalez@quironsalud.es (Ó.G.-L.); marta.lcastillo@fjd.es (M.L.-C.); andrea.kallmeyer@quironsalud.es (A.K.); ester.canovas@fjd.es (E.C.); 4 Department of Medicine, Autónoma University, 08193 Madrid, Spain; jegido@quironsalud.es (J.E.); olorenzo@fjd.es (Ó.L.); 5 Department of Cardiology, Hospital Universitario de Móstoles, 28935 Madrid, Spain; nieves.tarin@salud.madrid.org; 6 Department of Cardiology, Hospital de Fuenlabrada, 28942 Madrid, Spain; carmen.cristobal@salud.madrid.org (C.C.); dr.gutierrez@gmail.com (C.G.-L.); 7 Department of Cardiology, Rey Juan Carlos University, Alcorcón, 28933 Madrid, Spain; llopez@fhalcorcon.es; 8 Department of Cardiology, Hospital Universitario Fundación Alcorcón, 28922 Madrid, Spain; ahuelmos@yahoo.es; 9 Department of Cardiology, Hospital de Getafe, 28905 Madrid, Spain; joaquinjesus.alonso@salud.madrid.org; 10 Renal, Vascular and Diabetes Research Lab, IIS-Fundación Jiménez Díaz, 28040 Madrid, Spain; 11 Centro de Investigación Biomédica en Red de Diabetes y Enfermedades Metabólicas (CIBERDEM), Instituto de Salud Carlos III, 28029 Madrid, Spain

**Keywords:** C-reactive protein, MCP-1, NT-proBNP, inflammation

## Abstract

Clinical data indicate that patients with C-reactive protein (CRP) levels higher than 2 mg per liter suffer from persistent inflammation, which is associated with high risk of cardiovascular disease (CVD). We determined whether a panel of biomarkers associated with CVD could predict recurrent events in patients with low or persistent inflammation and coronary artery disease (CAD). We followed 917 patients with CAD (median 4.59 ± 2.39 years), assessing CRP, galectin-3, monocyte chemoattractant protein-1 (MCP-1), N-terminal fragment of brain natriuretic peptide (NT-proBNP) and troponin-I plasma levels. The primary outcome was the combination of cardiovascular events (acute coronary syndrome, stroke or transient ischemic event, heart failure or death). Patients with persistent inflammation (*n* = 343) showed higher NT-proBNP and MCP-1 plasma levels compared to patients with CRP < 2 mg/L. Neither MCP-1 nor NT-proBNP was associated with primary outcome in patients with CRP < 2 mg/L. However, NT-proBNP and MCP-1 plasma levels were associated with increased risk of the primary outcome in patients with persistent inflammation. When patients were divided by type of event, MCP-1 was associated with an increased risk of acute ischemic events. A significant interaction between MCP-1 and persistent inflammation was found (synergy index: 6.17 (4.39–7.95)). In conclusion, MCP-1 plasma concentration is associated with recurrent cardiovascular events in patients with persistent inflammation.

## 1. Introduction

Clinical and experimental data support an essential role for inflammation in atherothrombosis [1,2]. Inflammation contributes to the genesis and progression of atherosclerotic plaque development from the initial steps to plaque rupture, which underlies many acute ischemic events. Several inflammatory-related mechanisms are involved in various aspects of atherosclerosis, including immune cell recruitment, cell adhesion, collagen and matrix degradation, smooth muscle cell proliferation, platelet aggregation and thrombus formation [3]. From a clinical point of view, different emerging inflammatory biomarkers have been tested to identify individuals at risk for the first and recurrent cardiovascular events. Of these, acute phase C-reactive protein (CRP) is a circulating pentraxin that induces a number of activities in cells and tissues involved in the processes of atherothrombosis [4]. CRP is also a stable plasma biomarker for systemic inflammation. Blood levels of CRP depend on interleukin 6 and other inflammatory proteins and cytokines that activate its production in alveolar macrophages, hepatocytes, monocyte-derived macrophages and lymphocytes in atherosclerotic plaque [5].

It has been extensively demonstrated that CRP levels are associated with an increased risk of cardiovascular events, independently of cholesterol levels [6,7]. Thus, the Monitoring of Trends and Determinants in Cardiovascular Disease (MONICA) study demonstrated that elevated levels of CRP were associated with an increased risk of coronary events in healthy middle-aged men [8]. In addition, CRP levels also predicted cardiovascular risk in women [7]. Clinical guidelines recommend the use of CRP as a part of global risk prediction and suggest that levels of CRP of <1, 1 to <3, and ≥3 mg/L can be used to represent low, moderate, and high vascular risk [9]. Nowadays, it is considered that patients with CRP levels higher than 2 mg/L are on persistent inflammation [10].

Additional biomarkers may refine risk due to inflammation after a coronary syndrome. For this reason, we tested whether the combination of CRP concentrations with other biomarkers could potentially help to predict risk in patients with persistent inflammation. Thus, we selected a panel of biomarkers associated with higher risk of cardiovascular (CV) events [11], including monocyte chemoattractant protein-1 (MCP-1) and galectin-3, both involved in inflammation and atherosclerosis [12,13]; N-terminal fragment of brain natriuretic peptide (NT-proBNP), which is related with heart failure (HF); and Troponin I, a marker of myocardial injury. Based on CRP concentrations, 917 patients with stable coronary artery disease (CAD; those who suffered a previous acute coronary syndrome or myocardial infarction 6 to 12 months before) were divided in two groups, low (CRP < 2 mg/L) or persistent (CRP ≥ 2 mg/L) inflammation.

## 2. Results

From 917 patients with acute coronary syndrome, 574 had low inflammation (CRP < 2 mg/L) and 343 had persistent inflammation (CRP ≥ 2 mg/L). The clinical and demographic characteristics of the studied population according to CRP concentrations are summarized in Table 1. There were significant differences among groups with respect to body mass index, smokers and patients previously diagnosed with hypertension, diabetes and heart failure. Patients with persistent inflammation had higher levels of total cholesterol, LDL-c and triglycerides. They had also diminished GFR and showed higher percentages of coronary artery bypass and vessels affects and had less complete revascularization and previous acute ischemic events. Finally, these patients were more often under treatment with acenocumarol, oral antidiabetic drugs, diuretics and nitrates.

With respect to the biomarkers analyzed, NT-proBNP and MCP-1 plasma levels were increased in patients with persistent inflammation compared with those with low inflammation. No changes were observed in Tn-I or Galectin-3 plasma levels between patients with low or persistent inflammation.

### 2.1. Biomarkers and Primary Outcome

We focused our analysis on the role of NT-proBNP and MCP-1 plasma levels as predictors of outcomes, since these biomarkers were the only ones showing differences between patients with low or persistent inflammation. Primary outcome was determined from the day of examination onward, with a mean follow-up of 4.59 ± 2.39 years. During follow-up, 183 patients—95 with low and 88 with persistent inflammation—developed the primary end point. The univariate Cox proportional hazard models used are summarized in Tables S1 and S2. A multivariate Cox model was used to study the effects of variables that were statistically significant in the univariate analysis (Table 2). After adjustment, age (hazard ratio (HR): 1.03 (1.00–1.05); *p* = 0.028) was associated with an increased risk of developing the primary outcome in patients with low inflammation. In addition, patients taking statins or β-blockers showed a low risk for the outcome (HR: 0.43 (0.20–0.89), *p* = 0.023; HR: 0.54 (0.34–0.84), *p* = 0.007, respectively). No association was observed between NT-proBNP or MCP-1 plasma levels and the primary outcome in patients with low inflammation (Table 2).

In patients with persistent inflammation, the presence of atrial fibrillation (HR: 2.36 (1.04–5.33); *p* = 0.039), previous acute myocardial infarction (HR: 0.58 (0.34–0.99); *p* = 0.046) and complete revascularization (HR: 0.59 (0.34–0.99); *p* = 0.029) persisted as predictors of primary outcome after multivariate Cox analysis. In addition, patients taking statins showed a low risk for the outcome (HR: 0.48 (0.25–0.93); *p* = 0.029). However, patients receiving insulin showed a high risk for the outcome (HR: 2.14 (1.07–4.28); *p* = 0.031). Both NT-proBNP (HR: 1.22 (1.06–1.41); *p* = 0.007) and MCP-1 (HR: 1.26 (1.02–1.56); *p* = 0.032) plasma levels were associated with an increased risk of developing the primary outcome in patients with persistent inflammation.

### 2.2. MCP-1 and Acute Ischemic Events

To delimitate the roles of MCP-1 and NT-proBNP as cardiovascular risk biomarkers, secondary outcomes (acute ischemic events, heart failure or death) were analyzed. The predictors for acute ischemic events were studied in both patients with low and persistent inflammation. During the follow-up, 125 patients—61 with low and 54 with persistent inflammation—suffered an acute ischemic event. The univariate Cox proportional hazard models are summarized in Supplementary Tables S1 and S2. In the multivariate Cox analysis, no associations were observed between any variables analyzed and risk for acute ischemic events in patients with low inflammation.

LDL-c plasma levels (HR: 1.01 (1.00–1.02); *p* = 0.003) and insulin treatment (HR: 2.74 (1.32–5.70); *p* = 0.007) were associated with an increased risk of acute ischemic events in patients with persistent inflammation. In addition, patients taking statins (HR: 0.41 (0.20–0.84); *p* = 0.015) or with previous complete revascularization (HR: 0.49 (0.28–0.83); *p* = 0.009) were associated with a decreased risk for acute ischemic events. Interestingly, MCP-1 was the only biomarker that persisted as predictor for acute ischemic events after adjustment by confounders (HR: 1.31 (1.07–1.62); *p* = 0.010).

**Figure 1:**
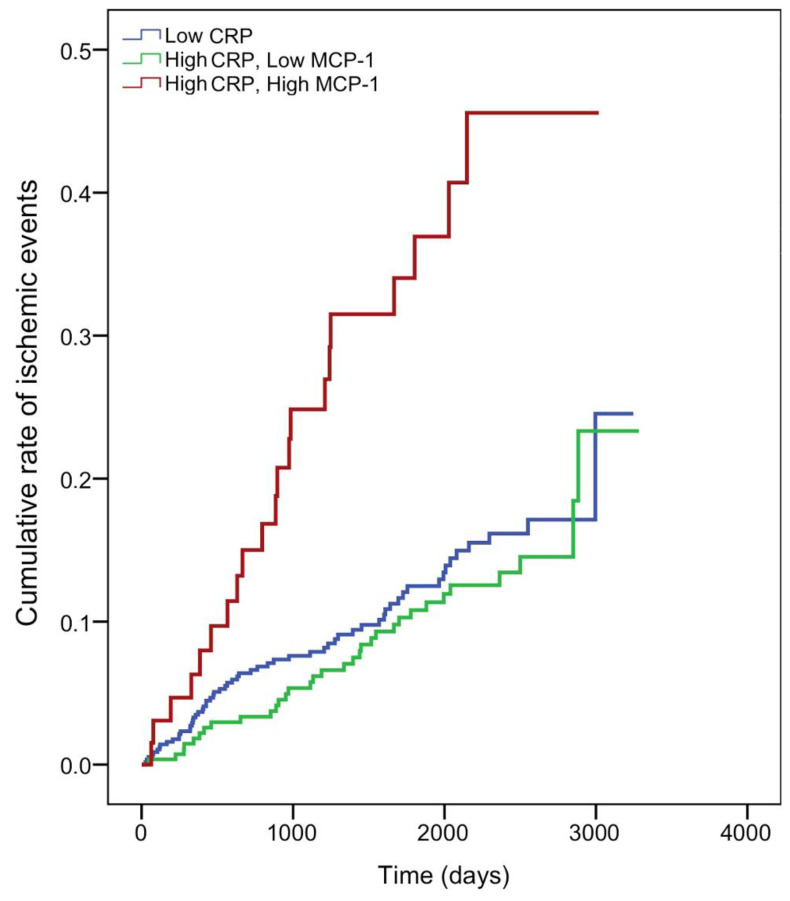
Kaplan–Meier plot of cumulative probability of an acute ischemic event when patients were grouped according to C-rective protein (CRP) and monocyte chemoattractant protein-1 (MCP-1) levels. CRP concentrations were defined as low (CRP < 2 mg/L) and high levels (CRP ≥ 2 mg/L). MCP-1 levels were defined as low MCP-1 (≤188 ng/L) or high MCP-1 (>188 ng/L), as determined by receiver operator characteristic curves.

Since both MCP-1 and CRP are proinflammatory, we explored the potential additive effects of CRP and MCP-1 in relation to outcome. MCP-1 cut-off value was identified using a receiver operating characteristic curve (ROC) and the Youden’s index, and was established at 188 ng/L. Thus, we divided the patients into three groups as follows: group 1 included patients with low inflammation (CRP ≤ 2 mg/L, *n* = 468) as a reference, group 2 included patients with persistent inflammation and low MCP-1 (*n* = 277) and group 3 included patients with persistent inflammation and high MCP-1 (*n* = 66). The Kaplan–Meier curve showed a significant association between high MCP-1 levels and persistent inflammation, with a reduction in the number of patients who remained event-free compared with those with low MCP-1 or patients with low CRP (log rank test 21.04; *p* < 0.001) (Figure 1).

In the univariate analysis, patients from group 3 had a significantly increased risk of ischemic events (HR: 2.68 (1.63–4.41); *p* > 0.001). Group 3 persisted as an independent predictor of ischemic events after adjustment by confounders (HR: 2.08 (1.25–3.47); *p* = 0.005) (Table 3).

Biologic interaction effects were calculated for crude and adjusted HR. In crude analysis, high MCP-1 levels showed a significant interaction (synergy index S = 12.3 (95% CI: 9.75–14.9)) with persistent inflammation. The relative excess risk due to interaction (RERI) was 2.98 (95% CI: 1.44–4.52). After adjustment for confounding factors, the S was 6.17 (95% CI: 4.39–7.95) and the RERI was 2.37 (95% CI: 1.08–3.66).

### 2.3. NT-proBNP and HF or Death

The predictors for HF or death were studied using univariate Cox analyses in both patients with low and persistent inflammation (Tables S1 and S2). During the follow-up, 96 patients—45 with low and 51 with persistent inflammation—suffered HF or death. After adjustment, age (HR: 1.08 (1.04–1.12); *p* < 0.001), LDL-c (HR: 0.98 (0.97–1.00); *p* = 0.034), taking antialdosterone antagonists (HR: 3.01 (1.12–8.08); *p* = 0.029) or β-blockers (HR: 0.40 (0.20–0.80); *p* = 0.029) was a predictor for HF or death in patients with low inflammation (Table 4). In addition, age (HR: 1.07 (1.03–1.10); *p* < 0.001), ejection fraction < 40% (HR: 2.60 (1.22–5.51); *p* = 0.012), HDL-c plasma levels (HR: 1.05 (1.02–1.08); *p* = 0.001) and treatment with insulin (HR: 3.22 (1.28–8.13); *p* = 0.013) were associated with an increased risk of developing HF and death in patients with persistent inflammation (Table 4). NT-proBNP plasma levels persisted as independent predictors of HF or death after adjustment in patients with either low or persistent inflammation (HR: 1.27 (1.03–1.57), *p* = 0.024; HR: 1.39 (1.16–1.66), *p* < 0.001, respectively) (Table 4).

## 3. Discussion

In this work, we investigated Tn-I, NT-proBNP, MCP-1 and Gal-3 plasma levels as predictors of recurrent cardiovascular events (acute ischemic events, heart failure and death) in patients with stable CAD and low or persistent inflammation. We observed that NT-proBNP and MCP-1 plasma levels were increased in patients with persistent inflammation. However, no changes were observed in Gal-3 or Tn-I levels, depending on the inflammatory status. Both MCP-1 and NT-proBNP plasma levels were associated with an increased risk of developing the primary outcome in patients with persistent inflammation. Our results also showed that NT-proBNP was associated with an increased risk of HF or death in patients with both low and persistent inflammation. More importantly, we showed that MCP-1 might be an additive biomarker for recurrent ischemic events only in patients with persistent inflammation.

MCP-1, a member of the C-C chemokine family, is synthetized by endothelial cells, smooth muscle cells and monocytes and macrophages within atherosclerotic plaques [14]. Oxidized LDL, different cytokines, homocysteine, angiotensin II, shear stress and other mediators of atherosclerosis induce MCP-1 production in vascular cells [15]. It is known that MCP-1, through its receptor C-C chemokine receptor type 2 (CCR-2) on monocytes, acts as a chemotactic factor to recruit monocytes into the vascular wall [16]. MCP-1 is highly expressed in human macrophage-rich atherosclerotic plaques [17]. The role of MCP-1 in atherosclerotic plaque development and progression has been extensively analyzed. Atherosclerosis is promoted when MCP-1 expression is increased in the carotid artery of rabbits [16]. Dominant-negative mutation of MCP-1 prevents vulnerable plaques from rupture in rabbits, independent of their lipid levels [18]. In addition, MCP-1 deletion reduced atherosclerosis progression in transgenic mice overexpressing human ApoB [19] and in low-density-receptor-deficient mice [20]. A significant reduction in atherosclerotic lesion size was also observed in CCR-2-deficient mice [21].

At a population level, MCP-1 plasma concentrations are positively correlated with different cardiovascular risk factors [22]. Higher MCP-1 plasma levels were observed in patients with stable coronary heart disease in the MONICA study [23] and with peripheral artery disease in the Atherosclerosis Risk in Communities study [24]. Elevated MCP-1 plasma levels have been also associated with an increased risk for myocardial infarction and death in patients with acute coronary syndrome [25]. In addition, MCP-1 concentrations provided independent prognostic value in the acute and chronic phases after acute coronary syndrome in the Aggrastat to Zocor trial (A to Z) [26]. Accordingly, we previously observed that MCP-1 is associated with cardiovascular events in patients with CAD [11]. In this study, we found that higher MCP-1 plasma levels were specifically associated with recurrent acute ischemic events in patients with systemic inflammation. The association of MCP-1 with acute ischemic events was strongest in patients with persistent inflammation, in whom high MCP-1 significantly predicted recurrent events. In fact, interaction between CRP and MCP-1 was observed even after adjusting for confounder factors. Thus, the HR for patients with both persistent inflammation and elevated MCP-1 levels was 6.17 times higher than the sum of the independent relative risk for each separately. These data indicates that MCP-1 levels offered prognostic information that was independent of that provided by CRP.

From a clinical point of view, reduction of the inflammatory response has been shown to be beneficial in decreasing cardiovascular events. Anti-inflammatory therapy targeting the interleukin-1β with canakinumab in the Canakinumab Antiinflammatory Thrombosis Outcome Study (CANTOS) study was associated with a significantly lower proportion of recurrent cardiovascular events independent of lipid levels in patients with acute myocardial infarction and CRP > 2 mg/L [27]. In addition, the Low Dose of Colchicine (LoDoCo) study showed that colchicine administered in addition to statins and other therapies appeared effective for the prevention of cardiovascular events (CVE) in patients with stable CAD [28]. In addition, the LoDoCo2 study demonstrated that the risk of cardiovascular events was significantly lower among those who received 0.5 mg of colchicine once daily than among those who received placebo [29]. Given the clear role of MCP-1 in atherosclerosis development and its complications, as well as the association of MCP-1 plasma levels with risk for future and recurrent acute ischemic events, MCP-1 should be further studied as a therapeutic target for the treatment of atherosclerosis. In fact, propagermanium, a drug used clinically for the treatment of chronic hepatitis in Japan, inhibits C-C chemokine receptor 2 function and reduces macrophage infiltration and atherosclerosis in Apolipoprotein E (ApoE) knockout mice [30]. This has recently been reported in a phase I dose escalation trial with propagermaium in patients with breast cancer [31] and a phase 2 trial with anti-MCP-1 (carlumab) in patients with idiopatic pulmonary fibrosis [32]. In addition, MLN1202, a specific humanized monoclonal antibody that interacts with CCR2-inhibiting MCP-1 binding, was used in a randomized, double-blind, placebo-controlled study in patients at risk for atherosclerotic cardiovascular disease (2 or more risk factors for atherosclerotic cardiovascular disease and circulating CRP >3 mg/L) [33]. MLN1202 was well tolerated and resulted in significant reductions in CRP. In addition, a randomized, double-blind, placebo-controlled phase 2a study to assess the effects of MLN1202 on atherosclerotic inflammation in subjects with stable atherosclerotic cardiovascular disease was started in 2015 (NCT02388971). However, this study was withdrawn during the recruitment phase for unknown reasons. Overall, if these therapies are proven to be safe and beneficial, investigation in atherosclerosis patients may be warranted.

Finally, NT-proBNP concentrations were associated with heart failure or death in both patients with low and persistent inflammation. These data are in accordance with several studies in which NT-proBNP concentrations were proven to be powerful diagnostic and prognostic biomarkers of heart disease [34].

Our study has some limitations. The use of the optimal cut-off point obtained for MCP-1 was used in combination with CRP in the same population, which could introduce a bias to our results. The limited size of patients with high MCP-1 and persistent inflammation may have influenced the results. Additionally, changes in MCP-1 during follow-up were not explored. However, the A to Z trial showed that MCP-1 levels did not change during follow-up after acute coronary syndrome [26]. Finally, data from the Biomarkers in Acute Coronary Syndrome & Biomarkers in Acute Myocardial Infarction (BACS-BAMI) database were used, however the findings reported here are different from those reported in previous papers [11,35].

In conclusion, our study demonstrates that MCP-1 plasma levels are independently associated with recurrent acute ischemic events in patients with stable CAD and persistent inflammation. In patients with CAD, the presence of both high CRP and MCP-1 levels could facilitate the decision for treatment with anti-inflammatory drugs.

## 4. Patients and Methods

### 4.1. Patients

The subjects of this study were 917 patients with stable CAD who had a previous acute coronary syndrome with or without ST-segment (ST) elevation 6–12 months before. These patients were part of the BACS-BAMI (Biomarkers in Acute Coronary Syndrome and Biomarkers in Acute Myocardial Infarction) project, developed in five different hospitals in Madrid (Spain), the inclusion and exclusion criteria for which were previously defined [11]. At baseline, clinical variables were recorded and plasma was withdrawn for analysis.

### 4.2. Ethics Statement

The research protocol conformed to the ethical guidelines of the 1975 Declaration of Helsinki, as reflected in a priori approval by the human research committees of the institutions participating in this study: Fundación Jiménez Díaz, Hospital Fundación Alcorcón, Hospital de Fuenlabrada, Hospital Universitario Puerta de Hierro-Majadahonda and Hospital Universitario de Móstoles (code 25/2007, 24 April 2007), as previously described [35]. All patients signed informed consent documents.

### 4.3. Study Design

Clinical variables were recorded at baseline. Patients were seen every year at their hospitals. The medical records were reviewed and patient status was confirmed at the end of follow-up. As previously described [35], the primary outcome was the combination of acute ischemic events (Non-ST acute coronary syndrome, ST-elevation myocardial infarction (NSTEACS, STEMI), stroke and transient ischemic attack) plus heart failure and all-cause mortality. The secondary outcomes were ischemic events and the composite of heart failure and death. Non-ST elevation acute coronary syndrome was defined as resting angina lasting more than 20 min in the previous 24 h, or new-onset class III-IV angina, along with transient ST depression or T wave inversion in the electrocardiogram considered diagnostic by the attending cardiologist or troponin elevation. ST elevation myocardial infarction was defined as symptoms compatible with angina lasting more than 20 min and ST elevation in two adjacent leads in the electrocardiogram without response to nitroglycerin, as well as troponin elevation. Past myocardial infarction was diagnosed in the presence of new pathological Q waves in the electrocardiogram, along with a concordant new myocardial scar identified either by echocardiography or nuclear magnetic resonance imaging. Stroke was defined as rapid onset of a neurologic deficit attributable to a focal vascular cause lasting more than 24 h or supported by new cerebral ischemic lesions in imaging studies. A transient ischemic attack was defined as a transient stroke with signs and symptoms resolving before 24 h without cerebral acute ischemic lesions at imaging techniques. HF was a clinical diagnosis made in accordance with practice guidelines. Events were adjudicated by at least two investigators of the study, along with a neurologist for cerebrovascular events. Although all events were recorded for each case, patients were excluded from the Cox regression analysis after the first event. Although the total number of events was also described, patients that experienced more than one event were computed only once for these analyses.

### 4.4. Biomarker and Analytical Studies

Plasma determinations were performed at the Laboratory of Nephrology at the Gómez-Ulla Hospital and at the Biochemistry Laboratory at Fundación Jiménez Díaz, as previously described [35]. The investigators who performed the laboratory studies were unaware of clinical data. Plasma concentrations of MCP-1 and galectin-3 were determined using commercially available enzyme-linked immunosorbent assay kits (BMS279/2, Bender MedSystems, Burlingame, CA, USA; DCP00, R&D Systems, Minneapolis, MN, USA, respectively) following the manufacturers’ instructions. Intra- and interassay coefficients of variation were 4.6% and 5.9% for MCP-1 and 6.2% and 8.3% for galectin-3, respectively. High-sensitive Troponin I (Hs-TnI) was assessed by direct quimioluminiscence (ADVIA Centaur, Siemens, Erlangen, Germany), High-sensitive C-reactive protein (Hs-CRP) protein was assessed by latex-enhanced immunoturbidimetry (ADVIA 2400 Chemistry System, Siemens, Erlangen, Germany) and NT-proBNP by immunoassay (VITROS, Ortho Clinical Diagnostics Raritan, West New York, NJ, USA). Lipids, glucose and creatinine determinations were performed using standard methods (ADVIA 2400 Chemistry System, Siemens, Erlangen, Germany). The estimated glomerular filtration rate (eGFR) was calculated using the Chronic Kidney Disease Epidemiology Collaboration (CKD-EPI) equation.

### 4.5. Statistical Analysis

Quantitative data with a normal distribution are presented as the mean ± standard deviation (SD) and were compared using the Student’s t test. Data that did not follow a normal distribution are displayed as the median (interquartile range) and were compared using the Mann–Whitney test. Qualitative variables are shown as percentages and were compared with the Chi-square or the Fisher’s test when appropriate.

The concentrations of MCP-1 and NT-proBNP are expressed in terms of a standardized variable as units of standard deviation (1-SD). The univariate Cox proportional hazard model was used to assess the variables associated with the outcomes. Then, a multivariate Cox hazard model was carried out, including those variables that achieved statistical significance in univariate analyses. Risk was estimated by including age, gender, diabetes, smoking status, hypertension, body mass index, history of cerebrovascular disease, atrial fibrillation, ejection fraction < 40%, complete revascularization, estimated glomerular filtration rate assessed by the CKD-EPI equation, lipid levels (low density lipoprotein-cholesterol (LDL-c), high density lipoprotein-cholesterol (HDL-c), triglycerides), therapy with anticoagulants, aspirin, statins, angiotensin-converting enzyme inhibitors, angiotensin receptor blockers, beta-blockers, diuretics, nitrates or nitroglycerin, complete revascularization and biomarkers NT-proBNP and MCP-1.

A receiver operator characteristic curve analysis was performed to determine the MCP-1 cutoff point in our patients for maximum sensitivity and highest specificity for prognosis of acute ischemic events based on Youden’s index. The Kaplan–Meier survival curve and long-rank test were used to compare time to outcome according to CRP and MCP-1 levels.

Finally, the presence of interactions between persistent inflammation and elevated MCP-1 levels and the effects on acute ischemic events were examined. HR rates with 95% CI were estimated for each category using CRP < 2 mg/L as the reference category. Following this, we calculated the relative excess risk due to interaction (RERI) and the synergy index (S) [36]. Analysis was performed with SPSS 19.0 (IBM, Armonk, NY, USA). Variables with *p* < 0.05 were considered statistically significant.

## Figures and Tables

**Table 1:** Characteristics of the studied population according to C-reactive protein (CRP) concentrations.

**Parameter**	**Patients with CRP < 2 mg/L (*n* = 574)**	**Patients with CRP ≥ 2 mg/L (*n* = 343)**	***p*-Value**
Age (year)	60 (52–71)	61 (51–72)	0.686
Male sex (%)	76.3	74.9	0.637
Body mass index (kg/m^2^)	27.7 (25.1–30.2)	28.7 (26.6–32.0)	**<0.001**
Smoker (%)	73.0	79.3	**0.032**
Hypertension (%)	61.0	68.2	**0.027**
Diabetes (%)	20.9	28.3	**0.011**
Previous heart failure (%)	9.6	15.2	**0.011**
Cerebrovascular event (%)	1.9	4.1	0.051
Present or past atrial fibrillation (%)	5.7	6.7	0.558
Total Cholesterol (mg/dL)	140 (121–158)	154 (134–172)	**<0.001**
LDL Cholesterol (mg/dL)	75 (63–89)	83 (68–99)	**<0.001**
HDL Cholesterol (mg/dL)	41 (35–48)	41 (36–47)	0.686
Triglycerides (mg/dL)	93 (72–125)	120 (89–167)	**<0.001**
Glucose (mg/dL)	101 (91–112)	100 (92–118)	0.243
GFR (CKD-EPI) (mL/min/1.73 m^2^)	81 (68–94)	77 (60–90)	**<0.001**
High-sensitivity C-reactive protein (mg/L)	0.5 (0.1–1.0)	4.1 (2.8–7.3)	**<0.001**
High-sensitivity troponin I (ng/L)	3.0 (0.0–9.0)	3.5 (0.0–12.0)	0.152
NT-ProBNP (ng/L)	165 (87–350)	188 (96–481)	**0.034**
MCP-1 (ng/L)	129 (103–171)	142 (111–180)	**0.006**
Galectin-3 (µg/L)	7.8 (6.0–9.7)	7.8 (5.8–10.0)	0.942
**Medical Therapy**			
Acetylsalicylic acid (%)	94.6	91.2	**0.049**
AntiP2Y12 (%)	78.9	67.6	**<0.001**
Acenocumarol (%)	4.2	7.9	**0.018**
Statins (%)	95.5	93.0	0.111
Oral antidiabetic drugs (%)	13.6	21.3	**0.002**
Insulin (%)	5.4	7.9	0.137
ACE inhibitors (%)	64.0	55.7	**0.013**
Angiotensin receptor blockers (%)	14.8	18.1	0.192
Aldosterone receptor blockers (%)	7.3	4.4	0.074
Betablockers (%)	79.6	77.2	0.399
Diuretics (%)	15.8	23.3	**0.005**
Nitrates (%)	11.5	17.2	**0.015**
**Data at Last Acute Coronary Event**			
STEMI/Non-STEACS (%)	53.0	42.0	**<0.001**
Number of vessels diseased	1.0 (1.0–2.0)	1.0 (1.0–2.0)	**0.001**
Coronary artery bypass graft (%)	5.6	11.1	**0.002**
Complete revascularization (%)	72.1	64.7	**0.019**

CRP, C-reactive protein; GFR, glomerular filtration rate; CKD-EPI, Chronic Kidney Disease Epidemiology Collaboration; NT-proBNP, N-terminal fragment of brain natriuretic peptide; MCP-1, monocyte chemoattractant protein-1; antiP2Y12, inhibitor of receptor for adenosine diphosphate (ADP) P2Y12; ACE, Angiotensin-converting enzyme; STEMI, ST-elevation myocardial infarction; Non-STEACS, non-ST elevation acute coronary syndrome.

**Table 2:** Multivariate Cox proportional hazard model for the incidence rates of acute ischemic events, heart failure and death.

**CRP < 2 mg/L**		
**Parameter**	**HR (95% CI)**	***p*-Value**
Age, years	1.03 (1.00–1.05)	**0.028**
Diabetes, yes	1.32 (0.65–2.66)	0.445
Hypertension, yes	1.64 (0.91–2.96)	0.098
History of CVE, yes	2.51 (0.97–6.45)	0.057
Ejection fraction < 40%, yes	1.00 (0.49–2.06)	0.997
CKD-EPI < 60 mL/min/1.73 m^2^	1.04 (0.59–1.82)	0.906
Statins, yes	0.43 (0.20–0.89)	**0.023**
ACE inhibitors, yes	0.77 (0.45–1.34)	0.364
ARB, yes	1.75 (0.93–3.29)	0.238
Antialdosterone, yes	1.58 (0.74–3.39)	0.238
β-Blockers, yes	0.54 (0.34–0.84)	**0.007**
Nitrates, yes	1.39 (0.83–2.31)	0.207
Diuretics, yes	0.81 (0.46–1.41)	0.456
Insulin, yes	1.40 (0.64–3.08)	0.401
Oral antidiabetic drugs, yes	1.34 (0.62–2.92)	0.458
NT-proBNP, 1-SD	1.14 (0.96–1.35)	0.144
MCP-1, 1-SD	1.07 (0.88–1.28)	0.493
Tn-I, 1-SD	1.05 (0.70–1.58)	0.815
**CRP ≥ 2 mg/L**		
**Parameter**	**HR (95% CI)**	***p*-Value**
Age, years	1.02 (1.00–1.04)	0.073
Sex, male	0.80 (0.48–1.32)	0.381
Hypertension, yes	0.93 (0.52–1.65)	0.803
Ejection fraction < 40%, yes	1.60 (0.88–2.92)	0.126
Atrial Fibrillation, yes	2.36 (1.04–5.33)	**0.039**
Acute myocardial infarction, yes	0.58 (0.34–0.99)	**0.046**
Complete Revascularization	0.59 (0.36–0.95)	**0.029**
LDL-c, mg/dL	1.01 (1.00–1.01)	0.091
HDL-c, mg/dL	1.02 (1.00–1.04)	0.081
Anticoagulants, yes	0.77 (0.34–1.74)	0.531
Statins, yes	0.44 (0.23–0.86)	**0.016**
Nitrates, yes	1.64 (0.93–2.90)	0.085
Diuretics, yes	1.04 (0.62–1.77)	0.874
Insulin, yes	2.14 (1.07–4.28)	**0.031**
NT-proBNP, 1-SD	1.22 (1.06–1.41)	**0.007**
MCP-1, 1-SD	1.26 (1.02–1.56)	**0.032**
Gal-3, 1-SD	1.16 (0.93–1.46)	0.192
Tn-I, 1-SD	1.18 (0.95–1.32)	0.136

CRP, C-reactive protein; HR, Hazard ratio; CVE, cardiovascular event; CKD-EPI, Chronic Kidney Disease Epidemiology Collaboration; ACE, Angiotensin-converting enzyme; ARB, angiotensin II recepotr blockers; NT-proBNP, N-terminal fragment of brain natriuretic peptide; MCP-1, monocyte chemoattractant protein-1; Tn-I, troponin I; Gal-3, galectin-3.

**Table 3:** Multivariate Cox proportional hazard model for the incidence of recurrent acute ischemic events in patients with CAD.

Parameter	HR (95% CI)	*p*-Value
Complete Revascularization	0.62 (0.43–0.91)	**0.014**
LDL-c, mg/dL	1.01 (1.00–1.01)	**0.022**
Statins, yes	0.45 (0.25–0.79)	**0.006**
Insulin, yes	2.32 (1.35–3.99)	**0.002**
CRP < 2 mg/L	1.00	
CRP ≥ 2 mg/L, MCP-1 ≤ 188 ng/L	0.79 (0.52–1.21)	0.280
CRP ≥ 2 mg/L, MCP-1 > 188 ng/L	2.08 (1.25–3.47)	**0.005**

HR, Hazard ratio; LDL-c, low-density lipoprotein-cholesterol; CRP, C-reactive protein; MCP-1, monocyte chemoattractant protein-1.

**Table 4:** Multivariate Cox proportional hazards model for the incidence of heart failure and death.

**CRP < 2 mg/L**		
**Parameter**	**HR (95% CI)**	***p*-Value**
Age, years	1.08 (1.04–1.12)	**<0.001**
Body mass index, kg/m^2^	0.97 (0.89–1.06)	0.498
Diabetes, yes	2.89 (1.01–8.28)	**0.048**
Hypertension, yes	1.61 (0.53–4.93)	0.402
History of CVE, yes	0.97 (0.19–4.95)	0.969
Ejection fraction < 40%, yes	1.06 (0.41–2.74)	0.906
LDL-c, mg/dL	0.98 (0.97–1.00)	**0.034**
CKD-EPI < 60 mL/min/1.73 m^2^	0.99 (0.44–2.25)	0.906
Anticoagulants, yes	1.60 (0.44–5.87)	0.475
Statins, yes	0.46 (0.14–1.50)	0.200
Antialdosterone, yes	3.01 (1.12–8.08)	**0.029**
β-Blockers, yes	0.40 (0.20–0.80)	**0.009**
Nitrates, yes	1.08 (0.50–2.36)	0.837
Diuretics, yes	1.27 (0.56–2.86)	0.563
Insulin, yes	1.33 (0.42–4.20)	0.630
Oral antidiabetic drugs, yes	1.00 (0.32–3.11)	0.997
NT-proBNP, 1-SD	1.27 (1.03–1.57)	**0.024**
MCP-1, 1-SD	1.13 (0.85–1.49)	0.140
Tn-I, 1-SD	1.33 (0.91–1.95)	0.140
**CRP ≥ 2 mg/L**		
**Parameter**	**HR (95% CI)**	***p*-Value**
Age, years	1.07 (1.03–1.10)	**<0.001**
Sex, male	0.99 (0.49–2.02)	0.980
Hypertension, yes	0.82 (0.36–1.88)	0.644
History of CVE, yes	2.11 (0.43–10.3)	0.358
Ejection fraction < 40%, yes	2.60 (1.22–5.51)	**0.012**
Atrial Fibrillation, yes	3.61 (1.28–10.1)	**0.015**
Complete Revascularization	0.76 (0.39–1.47)	0.413
HDL-c, mg/dL	1.05 (1.02–1.08)	**0.001**
CKD-EPI < 60 mL/min/1.73 m^2^	0.70 (0.35–1.41)	0.319
Anticoagulants, yes	0.56 (0.20–1.59)	0.279
Statins, yes	0.44 (0.17–1.16)	0.096
Antialdosterone, yes	0.55 (0.13–2.32)	0.414
Nitrates, yes	1.98 (0.89–4.43)	0.094
Diuretics, yes	1.11 (0.55–2.28)	0.765
Insulin, yes	3.22 (1.28–8.13)	**0.013**
NT-proBNP, 1-SD	1.39 (1.16–1.66)	**<0.001**
MCP-1, 1-SD	1.01 (0.74–1.38)	0.931
Gal-3, 1-SD	1.50 (0.93–3.08)	0.094
Tn-I, 1-SD	1.26 (0.90–1.43)	0.234

CRP, C-reactive protein; HR, Hazard ratio; CVE, cardiovascular event; CKD-EPI, Chronic Kidney Disease Epidemiology Collaboration; ACE, Angiotensin-converting enzyme; ARB, angiotensin II recepotr blockers; NT-proBNP, N-terminal fragment of brain natriuretic peptide; MCP-1, monocyte chemoattractant protein-1; Tn-I, troponin I; Gal-3, galectin-3.

## Data Availability

Not applicable.

## Supplementary Materials

The following are available online at https://www.mdpi.com/2077-0383/10/5/1137/s1: Table S1. Univariate Cox proportional hazards model in patients with Hs-CRP < 2 mg/L. Table S2. Univariate Cox proportional hazards model in patients with Hs-CRP ≥ 2 mg/L.
