# Supplementary material for: MCP-1 Predicts Recurrent Cardiovascular Events in Patients with Persistent Inflammation
Source: J Clin Med. 2021 Mar 9;10(5):1137. doi: 10.3390/jcm10051137 (PMC7963189; doi:10.3390/jcm10051137)
Supplement: Supplementary file 1 [file jcm-10-01137-s001.pdf]

Journal of Clinical Medicine

MDPI

Table S1. Univariate Cox proportional hazards model in patients with Hs-CRP < 2 mg/L.

	**Primary Outcome**		**Ischemic Events**		**Heart Failure/Death**	
**Parameter**	**HR (95%CI)**	***p*-Value**	**HR (95%CI)**	***p*-Value**	**HR (95%CI)**	***p*-Value**
Age, years	1.05 (1.03–1.07)	**<0.001**	1.02 (1.00–1.04)	0.055	1.11 (1.07–1.14)	**<0.001**
Sex, male	0.91 (0.58–1.44)	0.911	0.91 (0.51–1.61)	0.911	1.25 (0.65–2.39)	0.499
Smoker, yes	1.44 (0.95–2.19)	0.085	1.78 (1.07–2.97)	**0.027**	1.07 (0.56–2.04)	0.846
Body mass index, kg/m^2	1.00 (1.00–1.00)	0.951	1.00 (0.97–1.02)	0.946	0.95 (0.88–1.03)	0.202
Diabetes, yes	1.92 (1.25–2.96)	**0.003**	1.59 (0.92–2.77)	0.097	2.95 (1.63–5.37)	**<0.001**
Hypertension, yes	2.70 (1.62–4.52)	**<0.001**	2.29 (1.24–4.22)	**0.008**	4.56 (1.80–11.5)	**0.001**
History of CVE, yes	3.52 (1.43–8.69)	**0.006**	3.01 (0.94–9.63)	0.063	4.35 (1.34–14.0)	**0.014**
Ejection fraction <40%, yes	2.07 (1.19–3.60)	**0.010**	1.58 (0.75–3.32)	0.230	4.48 (2.31–8.72)	**<0.001**
Atrial Fibrillation, yes	1.23 (0.57–2.66)	0.598	0.82 (0.26–2.61)	0.733	2.05 (0.81–5.21)	0.131
Acute myocardial infarction, yes	0.74 (0.49–1.13)	0.163	0.53 (0.31–0.92)	**0.023**	1.22 (0.67–2.22)	0.512
Complete Revascularization	0.70 (0.46–1.06)	0.089	0.71 (0.42–1.19)	0.196	0.62 (0.34–1.14)	0.123
LDL-c, mg/dL	1.00 (1.00–1.01)	0.759	1.00 (0.99–1.01)	0.434	0.98 (0.97–1.00)	**0.037**
HDL-c, mg/dL	0.99 (0.97–1.01)	0.387	0.99 (0.96–1.01)	0.362	1.00 (0.97–1.03)	0.989
Triglycerides, mg/dL	1.00 (1.00–1.00)	0.493	1.00 (1.00–1.01)	0.063	0.99 (0.98–1.00)	0.076
CKD-EPI <60 mL/min/1.73 m^2	2.48 (1.58–3.87)	**<0.001**	2.19 (1.24–3.87)	**0.007**	3.36 (1.82–6.21)	**<0.001**
Acetylsalicylic acid, yes	0.68 (0.33–1.41)	0.306	1.22 (0.38–3.91)	0.732	0.51 (0.20–1.29)	0.154
AntiP2Y12, yes	0.71 (0.46–1.10)	0.115	0.97 (0.54–1.73)	0.909	0.68 (0.36–1.27)	0.231
Anticoagulants, yes	2.08 (0.96–4.50)	0.062	1.36 (0.43–4.35)	0.602	4.20 (1.77–9.95)	**0.001**
Statins, yes	0.31 (0.17–0.59)	**<0.001**	0.53 (0.21–1.31)	0.169	0.23 (0.10–0.52)	**<0.001**
ACE inhibitors, yes	0.62 (0.42–0.93)	**0.021**	0.51 (0.31–0.84)	**0.009**	1.08 (0.59–1.98)	0.800
ARB, yes	2.32 (1.49–3.63)	**<0.001**	2.62 (1.53–4.51)	**<0.001**	1.52 (0.75–3.09)	0.241
Anti-aldosterone, yes	2.17 (1.19–3.99)	**0.012**	1.53 (0.66–3.55)	0.326	5.31 (2.67–10.6)	**<0.001**
β-Blockers, yes	0.44 (0.29–0.66)	**<0.001**	0.56 (0.33–0.96)	**0.034**	0.28 (0.15–0.50)	**<0.001**
Nitrates, yes	2.26 (1.42–3.58)	**0.001**	2.19 (1.22–3.94)	**0.009**	2.58 (1.36–4.88)	**0.004**
Diuretics, yes	2.04 (1.31–3.19)	**0.002**	1.40 (0.76–2.58)	0.287	3.83 (2.11–6.70)	**<0.001**
Insulin, yes	2.53 (1.31–4.89)	**0.006**	2.71 (1.23–5.97)	**0.013**	3.03 (1.28–7.20)	**0.012**
Oral antidiabetic drugs, yes	1.69 (1.02–2.80)	**0.041**	1.25 (0.63–2.46)	0.518	2.19 (1.11–4.34)	**0.025**
NT-proBNP, 1-SD	1.40 (1.25–1.57)	**<0.001**	1.15 (0.96–1.37)	0.127	1.69 (1.48–1.91)	**<0.001**
MCP-1, 1-SD	1.27 (1.10–1.46)	**0.001**	1.08 (0.85–1.37)	0.545	1.41 (1.20–1.67)	**<0.001**
Gal-3, 1-SD	1.11 (0.99–1.25)	0.075	1.11 (0.95–1.29)	0.191	1.14 (0.98–1.33)	0.080
Tn-I, 1-SD	1.12 (0.93–1.35)	0.237	0.81 (0.41–1.58)	0.529	1.25 (1.05–1.48)	**0.010**

Table S2. Univariate Cox proportional hazards model in patients with Hs-CRP≥2 mg/L.

	Primary Outcome		Ischemic Events		Heart Failure/Death	
**Parameter**	**HR (95%CI)**	***p*-Value**	**HR (95%CI)**	***p*-Value**	**HR (95%CI)**	***p*-Value**
Age, years	1.04 (1.02–1.06)	**<0.001**	1.01 (0.99–1.04)	0.192	1.09 (1.06–1.12)	**<0.001**
Sex, male	0.63 (0.40–1.00)	**0.048**	0.64 (0.36–1.13)	0.640	1.84 (1.03–3.28)	**0.039**
Smoker, yes	1.60 (0.99–2.57)	0.054	1.61 (0.89–2.92)	0.118	0.58 (0.31–1.08)	0.084
Body mass index, kg/m^2	1.00 (0.99–1.02)	0.783	1.00 (0.97–1.02)	0.846	1.01 (0.99–1.02)	0.323
Diabetes, yes	1.12 (0.70–1.79)	0.630	1.49 (0.85–2.61)	0.162	1.00 (0.53–1.89)	0.999
Hypertension, yes	1.71 (1.04–2.79)	**0.033**	1.68 (0.90–3.14)	0.104	2.14 (1.07–4.28)	**0.032**
History of CVE, yes	1.69 (0.68–4.16)	0.257	0.94 (0.23–3.85)	0.930	3.42 (1.35–8.64)	**0.009**
Ejection fraction <40%, yes	2.54 (1.60–4.05)	**<0.001**	1.85 (0.99–3.45)	0.054	4.25 (2.40–7.53)	**<0.001**
Atrial Fibrillation, yes	3.62 (2.04–6.43)	**<0.001**	1.39 (0.50–3.85)	0.528	7.16 (3.77–13.6)	**<0.001**
Acute myocardial infarction, yes	0.63 (0.40–0.99)	**0.045**	0.57 (0.32–1.02)	0.057	0.84 (0.67–2.22)	0.564
Complete Revascularization	0.49 (0.32–0.75)	**0.001**	0.48 (0.28–0.81)	**0.007**	0.46 (0.26–0.80)	**0.007**
LDL-c, mg/dL	1.01 (1.00–1.01)	**0.028**	1.01 (1.00–1.02)	**0.004**	1.00 (0.99–1.01)	0.857
HDL-c, mg/dL	1.02 (1.01–1.04)	**0.004**	1.01 (0.99–1.03)	0.258	1.03 (1.01–1.05)	**0.008**
Triglycerides, mg/dL	1.00 (1.00–1.00)	0.560	1.00 (1.00–1.00)	0.276	1.00 (1.00–1.00)	0.841
CKD-EPI <60 mL/min/1.73 m^2	1.51 (0.96–2.38)	0.074	0.92 (0.48–1.74)	0.795	2.73 (1.56–4.78)	**<0.001**
Acetylsalicylic acid, yes	0.98 (0.47–2.02)	0.948	1.66 (0.52–5.33)	0.393	0.58 (0.26–1.30)	0.190
AntiP2Y12, yes	0.95 (0.61–1.48)	0.814	1.04 (0.58–1.84)	0.904	0.90 (0.50–1.62)	0.723
Anticoagulants, yes	2.61 (1.47–4.62)	**0.001**	1.26 (0.50–3.16)	0.624	3.98 (2.03–7.80)	**<0.001**
Statins, yes	0.38 (0.21–0.70)	**0.002**	0.35 (0.17–0.71)	**0.004**	0.41 (0.19–0.92)	**0.031**
ACE inhibitors, yes	0.98 (0.64–1.49)	0.914	0.82 (0.48–1.40)	0.474	1.19 (0.68–2.09)	0.545
ARB, yes	1.40 (0.85–2.31)	0.188	1.06 (0.53–2.11)	0.870	1.79 (0.96–3.32)	0.066
Anti-aldosterone, yes	2.16 (0.99–4.68)	0.052	1.88 (0.68–5.22)	0.227	2.69 (1.06–6.78)	**0.036**
β-Blockers, yes	0.91 (0.56–1.48)	0.708	1.23 (0.63–2.38)	0.543	0.71 (0.39–1.32)	0.181
Nitrates, yes	2.13 (1.33–3.42)	**0.002**	1.56 (0.82–2.97)	0.173	2.15 (1.16–4.01)	**0.016**
Diuretics, yes	1.69 (1.07–2.67)	**0.023**	1.22 (0.67–2.25)	0.514	2.70 (1.53–4.76)	**0.001**
Insulin, yes	3.02 (1.67–5.46)	**<0.001**	3.07 (1.50–6.28)	**0.002**	3.88 (1.88–8.02)	**<0.001**
Oral antidiabetic drugs, yes	0.97 (0.57–1.64)	0.898	1.24 (0.67–2.32)	0.494	1.00 (0.50–2.01)	0.989
NT-proBNP, 1-SD	1.37 (1.24–1.51)	**<0.001**	1.14 (0.94–1.40)	0.188	1.52 (1.38–1.68)	**<0.001**
MCP-1, 1-SD	1.38 (1.21–1.57)	**<0.001**	1.34 (1.12–1.60)	**0.001**	1.29 (1.11–1.50)	**0.001**
Gal-3, 1-SD	1.40 (1.14–1.72)	**0.001**	1.24 (0.95–1.62)	0.116	1.87 (1.45–2.41)	**<0.001**
Tn-I, 1-SD	1.17 (1.06–1.28)	**0.001**	1.23 (0.97–1.31)	0.120	1.23 (1.12–1.35)	**<0.001**
